# *ZBTB46*, *SPDEF*, and *ETV6*: Novel Potential Biomarkers and Therapeutic Targets in Castration-Resistant Prostate Cancer

**DOI:** 10.3390/ijms20112802

**Published:** 2019-06-08

**Authors:** AbdulFattah Salah Fararjeh, Yen-Nien Liu

**Affiliations:** 1 PhD Program for Cancer Molecular Biology and Drug Discovery, College of Medical Science and Technology, Taipei Medical University, Taipei 11031, Taiwan; d621104004@tmu.edu.tw; 2 Graduate Institute of Cancer Molecular Biology and Drug Discovery, College of Medical Science and Technology, Taipei Medical University, Taipei 11031, Taiwan; 3 TMU Research Center of Cancer Translational Medicine, Taipei Medical University, Taipei 11031, Taiwan

**Keywords:** androgen receptor (AR), castration-resistant prostate cancer (CRPC), zinc finger and BTB domain-containing protein 46 (*ZBTB46*), SAM pointed domain containing ETS transcriptional factor (*SPDEF*), ETS variant gene 6 (*ETV6*)

## Abstract

Prostate cancer (PCa) is the second most common killer among men in Western countries. Targeting androgen receptor (AR) signaling by androgen deprivation therapy (ADT) is the current therapeutic regime for patients newly diagnosed with metastatic PCa. However, most patients relapse and become resistant to ADT, leading to metastatic castration-resistant PCa (CRPC) and eventually death. Several proposed mechanisms have been proposed for CRPC; however, the exact mechanism through which CRPC develops is still unclear. One possible pathway is that the AR remains active in CRPC cases. Therefore, understanding AR signaling networks as primary PCa changes into metastatic CRPC is key to developing future biomarkers and therapeutic strategies for PCa and CRPC. In the current review, we focused on three novel biomarkers (*ZBTB46*, *SPDEF*, and *ETV6*) that were demonstrated to play critical roles in CRPC progression, epidermal growth factor receptor tyrosine kinase inhibitor (EGFR TKI) drug resistance, and the epithelial-to-mesenchymal transition (EMT) for patients treated with ADT or AR inhibition. In addition, we summarize how these potential biomarkers can be used in the clinic for diagnosis and as therapeutic targets of PCa.

## 1. Introduction

Prostate cancer (PCa) is the most common cancer expected to occur in men with an estimated 164,690 new cases annually in the US, and it is the second most common cause of cancer deaths in men after lung cancer [1]. It is responsible for approximately 300,000 deaths each year [2]. The five-year overall survival rate is 99%, which drops significantly to 30% in the distant stage. PCa mortality declined by 52% over the past two decades as a result of earlier detection, improved screening, and treatment advances. The first biomarker used for PCa screening was prostatic acid phosphatase (PAP) [3], the use of which was limited in the clinic once screening with the prostate-specific antigen (PSA) was initiated in 1979 [4].

PSA, which was first described in 1979, is produced by the prostatic epithelium and periurethral gland, and it can be detected in blood samples. Annual PSA screening was recommended for men over 50 years of age in the past two decades [5]. This recommendation has changed over the years because of the declines in early-stage PCa and PSA-based screening for those patients older than 50 years [6,7,8]. A randomized trial was conducted to determine whether PSA-based screening for PCa could decrease disease-related mortality [9]. The Prostate, Lung, Colorectal, and Ovarian (PLCO) Cancer Screening trial in the US with seven years of follow-up found no significant difference in the incidence or death rate for PCa between the PSA-tested group and the control group, indicating no benefit for patients who were screened for PSA. Furthermore, in 2012 the US Preventive Services Task Force (USPSTF) recommended against PSA testing based on several major trails of PSA screening and gave a grade D recommendation [10,11]. However, in 2017, the USPSTF upgraded PSA-based screening to grade C, which has created controversial decisions in urology databases [12].

Recently, several guidelines were developed to enhance PCa screening in place of the PSA test, decrease unnecessary biopsies, and increase the specificity for cancer detection. For example, according to the National Comprehensive Cancer Network (NCCN), in 2016, several biomarkers were recommended for screening before taking biopsies from men with PSA of >3 ng/ml such as the percent of free PSA, 4K score, and a prostate health index (PHI) [13]. Additionally, in 2016, the European Association of Urology (EAU)–European Society for Radiotherapy & Oncology (ESTRO)–International Society of Geriatric Oncology (SIGO) guidelines on PCa screening, diagnosis, and treatment strongly recommended testing PSA with a digital rectal examination (DRE) prior to prostate biopsies. If the PSA results are within 2–10 ng/mL and the DRE is normal, they recommend one of the following tests be performed: a risk calculator or PHI, 4K score, PCa gene (*PCA3*), *HOXC6*/*DLX1*, or imaging [14].

Treatment options for PCa include a prostatectomy, radiotherapy, chemotherapy, hormonal therapy, and even active surveillance (AS). In AS treatment, patients require no radiation, surgery, or chemotherapy. Patients undergo a conservative management approach such as routine screening to monitor the progression of cancer for those with a low risk or men with a Gleason score of ≤6 and a PSA level of <10 ng/mL [15,16]. However, many patients in the late stage of PCa with metastatic lesions to other parts of the body are more resistant to all treatment strategies including chemotherapy and hormonal therapy [17,18].

Normally, the androgen hormones, testosterone and dihydrotestosterone (DHT), are important for prostate function and growth in males. The androgen receptor (AR) is expressed by luminal prostate cells [19]. However, PCa cells express high levels of the AR with excess activation of AR signaling pathways, which enhances cellular proliferation and tumor formation [20,21,22]. Androgens are steroidal hormones that are produced at high levels in males and at low levels in females. In men, androgens regulate, stimulate, and control sexual characteristics, including the growth, development, and function of the prostate. Testosterone is the most abundant circulating hormone in men and is mainly produced by the testes [23,24]. Actions of androgens are mediated by binding to the AR, which are then translocated to nuclei where they regulate various gene expressions.

Despite the heterogeneity of the etiology and progression of PCa, the influence of androgen signaling on PCa seems to include all aspects of the disease. Therefore, androgen deprivation therapy (ADT) is the key regimen for patients with advanced PCa [25,26]. Nevertheless, the majority of patients eventually relapse and become resistant to ADT, which leads to increased PSA levels, increased tumor sizes, and metastasis to other organs with disease-related symptoms within two to three years [27]. This situation is referred to as metastatic castration-resistant PCa (CRPC) [28].

Several mechanisms have been proposed to explain the development of CRPC [27,29,30,31,32,33], among which reactivation of the AR pathway in many cases of CRPC remains an important mediator in the continuum of CRPC [34]. Therefore, understanding AR’s function before and after ADT and the mechanisms that cause metastatic CRPC are emerging needs for developing novel therapeutic strategies.

## 2. Standard Biomarkers of Prostate Cancer (PCa)

Nowadays, screening and diagnosis of PCa involve a physical examination. A digital rectal examination (DRE) is considered the primary assessment test for the prostate [35], while measuring serum concentration of PSA is an inexpensive test and is used for early detection and prognosis of PCa [36]. To increase the specificity of PSA, the PSA density (PSAD), which is the PSA (ng) divided by the prostate volume (mL), is evaluated [37]. In addition, the most common diagnostic procedure for PCa is transrectal ultrasonography (TRUS), which enables an early diagnosis of PCa and is considered a highly sensitive method [38]. In clinical practice, if the PSA is elevated and there is a positive result for the DRE, further tests are usually performed for a diagnosis or to exclude PCa. A TRUS-guided prostate biopsy is often done to collect a tissue sample from the prostate. Several predictive tools have been developed for the diagnosis and prognosis of PCa. Recently, a new study suggested that magnetic resonance imaging (MRI) could reduce unnecessary biopsies for those patients with suspected PCa. That study was conducted to evaluate MRI-based prostate cancer detection, which applied MRI, MRI-TRUS fusion-guided biopsies, and 12 core systemic biopsies in all patients. They found that using an MRI scan first to decide the need for a biopsy could reduce biopsies by 38% [39].

The only biomarkers for PCa that have been approved in clinics by the US Food and Drug Administration are the total and free PSA [40]. PSA has been widely used for PCa screening and diagnosis [41]; however, as we mentioned earlier, it lacks specificity as a result of several conditions, such as prostatic infection, when the PSA level might also increase [42]. Therefore, establishing a specific biomarker or panel of markers for PCa is a major challenge for scientists and physicians because PCa is a silent disease with no signs or symptoms presenting until it has reached an advanced stage or has metastasized to other parts of the body.

Recently, several proposed biomarkers for PCa were identified in either tissues or body fluids. For example, α methylacyl coenzyme A racemose (AMACR) was detected in PCa tissues [43]; an immunohistochemical (IHC) analysis with an anti-AMCAR/P504S antibody is considered a reliable test to screen for PCa in pathological specimens based on several reports, as AMACR was detected in more than 80% of malignant tissues [44,45,46]. Xu et al. used complementary (c)DNA, established from normal prostate and pancreatic tissues in addition to prostate tumor tissues, and a microarray analysis to identify differentially expressed genes in human PCa. Those authors found that AMCAR/P504S was specifically overexpressed in prostatic adenocarcinomas and was undetected in benign prostate tissues [47]. This result was confirmed by Luo et al. who found that AMCAR was nine-fold upregulated in PCa compared to normal tissues by analyzing messenger (m)RNA levels [48].

Bone turnover biomarkers (BTMs) are a group of proteins and their derivatives that are expressed during bone remodeling activities. PCa was reported to metastasize to bone, and this will lead to the involvement of several bone biomarkers during bone metabolism in PCa. Therefore, measuring BTMs in either the serum or tumor tissues can provide a new platform for PCa screening and prognosis [49]. For example, BTMs such as alkaline phosphatase (ALP), octeocalcin [50], and hepsin are all overexpressed in PCa tissues [51,52]. More than 80% of PCa patients die from bone metastasis [53], which raises the levels of bone markers such as ALP and octeocalcin [54]. Moreover, high levels of octeocalcin were detected in nonmetastatic prostate epithelial tumor cells at the RNA but not the protein level. However, higher protein levels of octeocalcin were detected in metastatic bone-derived PCa [55].

Hepsin is a member of type II transmembrane serine proteases (TTSPs), which are thought to play key roles in cell growth and tumor invasion by breaking down membranes. Overexpression of hepsin was reported in several solid tumors, including PCa, and was thus suggested as a biomarker for PCa [56,57,58]. Therefore, several classes of hepsin inhibitors were reported, such as indolecarboxamidines and benzamidines [57]. However, exploring novel biomarkers that can be used for diagnosis, screening, or tracking of the progression of PCa from primary to metastatic disease corresponding to the AR or ADT is urgently needed.

## 3. Androgen Receptor (AR) and PCa

The AR gene is located on X-chromosome q11-12 and consists of eight exons that code about a 111-kDa protein. The protein contains three major functional domains: an N-terminal domain (NTD) encoded by the first exon (residues 1–555), a DNA-binding domain (DBD) encoded by exons 2 and 3 (residues 556–623), and a ligand-binding domain (LBD) encoded by exons 4–8 at the C-terminal end (residues 665–919) [59,60,61]. The AR, also called nuclear receptor subfamily 3, group C, gene 4 (*NR3C4*), is considered to be a nuclear receptor transcriptional factor (TF) [62] and belongs to the steroid hormone nuclear receptor family. The AR is a ligand-dependent TF that is activated by binding to androgenic hormones, testosterone, or DHT, and then the AR is translocated to the nucleus where it regulates a wide range of target genes [63]. By transactivation of the AR in nuclei, several downstream signals are activated that influence the growth, development, and maintenance of the prostate gland [64].

The human prostate is mainly organized into an epithelial bilayer of cells: basal and luminal epithelial cells. There are also some neuroendocrine (NE) cells, which are less understood [65]. Luminal cells are protein-secretory cells that surround the lumen and are characterized by high expressions of the AR, cytokeratin 8 (*CK8*), CK18, and *NkX3.1*. On the other hand, basal cells make up a layer that supports luminal cells and is characterized by *CK5* and *CK14* with a low or undetectable level of the AR [66]. It was reported that both types of epithelial cells were self-sustaining in adult murines according to a lineage-tracing method [67,68,69]. Moreover, the plastic property of basal cells can generate luminal cells under stimulation by embryonic-derived urogenital sinus mesenchymal (UGSM) cells during prostate development. In androgen deprivation conditions, most luminal cells undergo apoptosis, with no such effect occurring to basal cells, resulting in regression of prostate size. Androgen treatment can return the prostate size to normal and cause luminal cells to regenerate, indicating that androgen mediates both prostate regression and regeneration. Development of the epithelium depends on paracrine signaling produced in the mesenchyme in response to androgen-AR binding. Using tissue recombination approaches and a murine AR-knockout model, researchers revealed an interaction between the epithelium and mesenchyme that mediated AR activities [70]. Recent reports suggested a role of the stromal but not epithelial AR in direct prostate development through modulation of several growth factors such as insulin-like growth factor [71,72], fibroblast growth factor [73], and vascular endothelial growth factor [74]. A decrease in prostate development was observed with AR knockout in fibroblast-specific protein 1 Cre AR-knockout (FSP-ARKO) mice [75].

It is believed that human PCa originates from luminal cells because it mostly has luminal epithelial phenotypes, which are characterized by high levels of AR and an absence of basal epithelial cells [76,77]. Several studies on murine models showed that both basal and luminal cells can initiate PCa in an organoid culture system [69,78].

NE cells are scattered and arise from epithelial stem cell units [79]. The number of NE cells varies in PCa, with some prostatic tumor tissues containing abundant NE cells [80,81]. Several markers were identified for localizing cancerous NE cells by IHC staining, such as chromogranin A, synaptophysin, and neuron-specific enolase [82]. NE PCa cells are considered an aggressive type and are characterized by low to nil AR [83]. In a xenograft animal model, the percentage of NE-differentiated (NED) cells significantly increased after castration [84,85]. NE PCa (NEPC) is more likely to become established and grow PCa cells in an androgen-deprived environment; for example, the growth of xerographic LNCaP cells from a castrated host were associated with the presence of NE cells, which may have activated the AR in an androgen-deprived environment and, thus, promoted tumor growth [86]. NEPC was demonstrated to be resistant to ADT and displayed a high metastatic PCa propensity with average survival of less than a year [87].

In the absence of testosterone or DHT, the AR is stabilized by heat shock protein (HSP)-90, HSP-70, and HSP-56 as well as cytoskeletal proteins [88,89] in the cytoplasm. A cytoskeletal protein, filamin A (FlnA), was reported to interact with the AR at the Hing-region. Researchers used FlnA-deficient cells that inhibited the transactivation of the AR to nuclei even after long exposure to a synthetic ligand, which indicated modulation of AR’s activity and action by FlnA [90]. In the presence of androgen, the association between the AR and FlnA increased, which activated Rac1 and focal adhesion kinase (FAK) to enhance the cells’ migratory ability and possibly affect PCa progression [91]. A recent report demonstrated involvement of the AR target gene—alpha-2-glycoprotein 1, zinc-binding (*AZGP1*)—in induction of PCa proliferation and metastasis. The authors revealed that AR signaling was responsible for upregulation of *AZGP1* and enhancement of cell proliferation in vitro and in vivo through the androgen/AR axis [92].

Therefore, any interruption or mutation of the AR gene or androgen-regulated pathways may affect the growth and development of the prostate and lead to PCa progression. Like normal prostate cells, PCa cells also require the AR to grow and survive. Androgen and the AR regulate the ratio between cell proliferation and cell death, which is higher in PCa and results in continuous cell proliferation and growth [93].

## 4. Mechanism of Castration-Resistant Prostate Cancer (CRPC)

Both ADT and androgen-suppression therapy are hormonal manipulation therapies aimed at preventing PCa progression by reducing testosterone or DHT levels or by blocking AR signaling pathways [94,95]. Furthermore, ADT has been extended to adjuvant and neoadjuvant settings for radiation and surgical therapies. Although ADT has demonstrated benefits in almost all men with advanced or metastatic PCa, most patients become resistant to hormonal treatment within three years and exhibit progression and growth of PCa to hormonal-refractory PCa or androgen-independent PCa. These terms were replaced with CRPC because patients were unable to respond to castration treatment [96]. Recently, more than 40 experts in the PCa field discussed definitions and criteria that needed to be included in a diagnosis of CRPC. They decided that CRPC should meet two important criteria: 1) a testosterone level in a castrated patient of <1.7 nmol/L, and 2) monitoring of the biochemical progression by the PSA level needs to be increased to two or more than three times a week with a >50% increase of the lowest or more than two measured increases in lesions [97].

During the progression of PCa from primary to CRPC, several changes occur to the AR and AR signaling pathway that could provide an explanation for the CRPC mechanism. The most common genetic change in CRPC patients is AR gene amplification [98], which accounts for more than 80% of CRPC patients. A further study based on the fluorescence in situ hybridization (FISH) technique demonstrated that no AR amplification was observed in benign prostate hyperplasia, and just 2% was detected in primary PCa tumors. However, more than 20% of CRPC tumors exhibit AR amplification [99]. In patients with AR overexpression, PCa cells can survive and grow even under ADT and progress to metastatic CRPC. It was reported that retinoblastoma (RB) expression was decreased in metastatic PCa and CRPC, and this was associated with poor clinical outcomes. Those authors concluded that perturbation of the RB-E2F1-AR axis in CRPC was sufficient to induce AR overexpression and progression to the lethal phenotype of PCa [100].

Several AR point mutations have been identified, which enhance the AR’s activity even at low levels of androgen. Most AR mutations occur in the NTD and/or LBD regions [101,102]. For example, the most common mutation of the AR is T878A, which leads to loss of agonist specificity and improves the sensitivity for progesterone and estrogen, thereby activating the AR [103]. Several mutations, such as T878A, H875T/Y, and W742C, exist in 15% of CRPC cases and can be detected in the circulating free DNA of patients with CRPC. These mutations could be used as biomarkers or for precision medicine in CRPC patients [104].

A biomarker is defined as a “characteristic that is objectively measured and evaluated as an indicator of normal biologic processes, pathogenic processes, or pharmacologic responses to a therapeutic intervention" [105]. However, a measurement that can be used in therapeutic trials as a substitute for a clinical end point, such as overall survival, is called a surrogate biomarker [106]. Recently, several therapeutic biomarkers and targets for CRPC were proposed that could become clinically valuable and could alter or inform medical decisions. In the current review, we focused on several potential biomarkers of CRPC and discussed their utility in clinical and research profiles.

## 5. Zinc Finger and BTB Domain-Containing Protein 46 (*ZBTB46*)

*ZBTB46*, also known as *BTBD4*, *zDC*, and *BZEL*, is a TF that belongs to the POZ and Krüppel (POK)/zing finger and BTB (ZBTB) protein family [107]. It was found that *ZBTB46* was specifically and selectively expressed by classical dendritic cells (DCs; cDCs), but not by other immune cells such as macrophages, monocytes, or cells of a lymphoid or myeloid lineage, and it maintains cDCs in a quiescence state [108,109]. This specific expression of *ZBTB46* makes it a useful biomarker for clinical diagnoses of DC malignancies [108]. Satpathy et al. [108] examined expression levels of *ZBTB46* by IHC staining in patients with histiocytic disorders, which are human malignancies of DCs and related myeloid cell types [110]. *ZBTB46* showed a clear DC lineage identity.

Outside of the immune system, *ZBTB46* is also expressed in quiescent endothelial cells (ECs). Using in vitro and in vivo systems, *ZBTB46* was found to inhibit EC proliferation, and overexpression of *ZBTB46* in an EC line led to decreased cell proliferation by increasing the G_0_-G_1_ cell population, which was characterized by low Ki67 expression. Furthermore, overexpression of ZBTB46 caused decreased expressions of several cell cycle genes, viz., cyclins and cyclin-dependent kinases (CDKs), and increased p21 but not p27 [111]. Normally, *ZBTB46* regulates expressions of several genes by binding to the gene zinc finger-binding domain and mediates chromatin remodeling and transcriptional regulation [112].

Recently, Chen et al. identified *ZBTB46* as a novel tumor promoter in PCa, which was negatively regulated by AR signaling via microRNA-1 (miR-1)-mediated downregulation [113]. Androgen hormones activate the AR and miR-1 signaling pathway, which suppresses *ZBTB46*. In PCa cells, in response to *ZBTB46* knockdown, only Snail was reduced, while E-cadherin expression was enhanced. However, overexpression of *ZBTB46* enhanced expression of Snail and decreased E-cadherin levels. Since Snail was shown to have a role in the EMT and metastasis in PCa [114,115,116], a study demonstrated that overexpression of *ZBTB46* promotes AR-independent proliferation [113].

In parallel, the role of *ZBTB46* in promoting NEPC progression and its association with inflammatory responses were elucidated [117]. Chen et al. showed that in ADT-treated PCa, *ZBTB46* levels were enhanced by loss of the expression of the SAM pointed domain-containing ETS transcriptional factor (*SPDEF*), which is believed to act as a tumor suppressor gene and to be regulated by AR signaling [118]. A low level of *SPDEF* leads to an increase in the expression of *ZBTB46*, thus facilitating an abundance of prostaglandin endoperoxide synthase 1 (*PTGS1*), which was reported to participate in inflammation, arthritis, and cancer pathophysiology [119,120,121,122]. Those studies demonstrated that *ZBTB46* acts as a transcriptional coactivator and induces *PTGS1* expression. Increasing the *PTGS1* level contributes to NE differentiation of PCa cells following ADT. *ZBTB46* activation of the AR inhibits PCa, resulting in NEPC differentiation and an abundance of *PTGS1* in PCa patients. Overall, *ZBTB46* can be used as a new novel biomarker and therapeutic target for PCa and NEPC.

**Figure 1:**
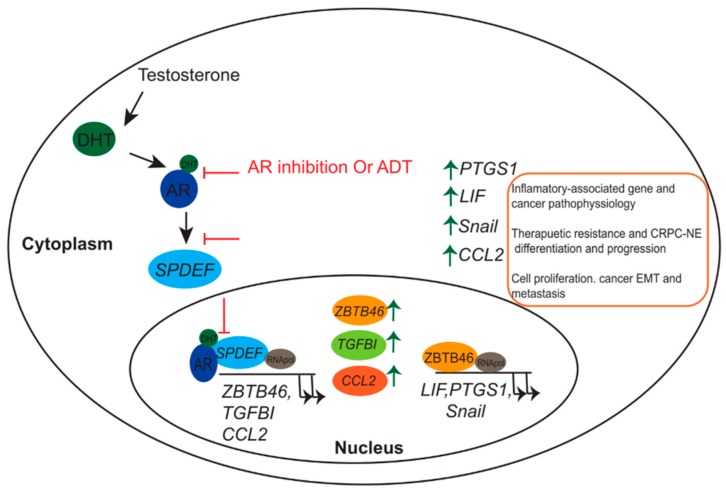
Schematic representation of androgen deprivation therapy (ADT) or androgen receptor (AR) antagonist in mediating therapeutic resistance and neuroendocrine (NE) differentiation of prostate cancer (PCa). Inactivates of the AR–SAM pointed domain-containing ETS transcriptional factor (*SPDEF*) signaling pathway via AR antagonist or by ADT induce the expression of *ZBTB46*, *TGFBI,* and *CCL2* (**Left**). *ZBTB46* acts as a transcriptional inducer for *LIF*, *PTGS1,* and *Snail,* which further enhance tumor progression, metastasis, therapeutic resistance, and castration-resistant PCa (CRPC)-NE differentiation of PCa (**Right**).

More recently, *ZBTB46* was shown to be involved in induction of the leukemia inhibitory factor (LIF)-signal transducer and activator of transcription 3 (STAT3) signaling pathway in ADT PCa cells [123]. LIF is a member of the interleukin (IL)-6 class and protects neurons and oligodendrocytes from oxidative stress [124]. In addition, the LIF was shown to activate the Janus kinase (JAK)/STAT3 signaling pathway, which promotes the differentiation of glial nerve sheath cells and their migration [125]. LIF overexpression was reported in breast, pancreatic, and prostate cancers, and it activated JAK/STAT3, which promoted cell tumorigenicity [125,126,127]. Since LIF plays a key role in NE cell differentiation, there is a high level of LIF in advanced stages of cancers, Liu et al. investigated the molecular mechanism of LIF in CRPC and NE differentiation during ADT. Levels of the LIF and NE markers were higher in the AR-suppressed PC3 PCa cell line, the RasB1 cell line (an aggressive cell line), and in small-cell NE carcinoma (SCNC). Furthermore, treating undifferentiated cells with the LIF protein or stably expressing the LIF led to induction of LIFR/STAT3 and NE markers. In addition, IHC staining of paraffin-embedded tissue samples from PCa patients showed an increase in the nuclear level of *ZBTB46* and an association with cytoplasmic LIF, which was observed in high-grade patients. *ZBTB46* regulates LIF expression by directly binding to the LIF-regulatory sequence, which then activates the LIF/STAT3 signaling pathway in ADT patients and facilitates NE differentiation [123] (Figure 1).

## 6. SAM Pointed Domain-Containing ETS Transcriptional Factor (*SPDEF*)

*SPDEF* is an E26 transformation-specific (ETS) TF [128] and has several functions, such as mediating protein–protein interactions, RNA-binding, and lipid molecule interactions, in addition to its transcriptional activities [129]. An analysis of mRNA levels of *SPDEF* in normal human tissues indicated that the highest levels were detected in prostate tissues and the salivary gland [130]. A number of reports suggested that *SPDEF* should be considered a tumor-suppressor gene in PCa [131]. Loss of *SPDEF* in PCa is associated with worse clinical outcomes and poor differentiation [132,133]. Knockdown of *SPDEF* led to enhanced prostatic cell migration, invasion, and metastasis [133,134]. On the other hand, ectopic expression of *SPDEF* efficiently suppressed the in vitro and in vivo metastasis of PCa [133,134]. It was reported that *SPDEF* was regulated by the AR pathway in PCa, and downregulation of *SPDEF* may be involved in the EMT of PCa in response to ADT. Recently, Tsai et al. reported a mechanistic link between ADT and the EMT in a subset of ADT-resistant PCa cells, where the chemokine C-C motif ligand 2 (*CCL2*) was stimulated through inactivation of AR-mediated *SPDEF* [135]. In that study, several approaches were performed to investigate the feasible correlation between AR–*SPDEF*–*CCL2* in PCa and metastatic CRPC. An inverse correlation was observed between SPFDEF and CCL2 after ADT compared to the intensity of both proteins before ADT. Low *SPDEF* and high *CCL2* levels after ADT were detected using IHC and tissue samples from PCa patients. Once the AR is activated, the *SPDEF* expression level is enhanced, and it is translocated to nuclei to suppress *CCL2* expression after binding to the *CCL2* promoter region, consequently inhibiting the EMT in PCa. However, in CRPC, AR inhibition by ADT leads to suppression of *SPDEF* expression and enhancement of *CCL2* and the EMT. This novel model could help in predicting therapeutic outcomes after ADT or standard PCa regimens [136].

A previous report demonstrated that transforming growth factor (TGF)-β signaling acts as a driver for activation of the AR in ADT treatment of CRPC [137]. Moreover, TGF-β signaling regulates the transcriptional activity of *SPDEF* [138]. Recently, Chen et al. proposed a novel model indicating the role of the AR–*SPDEF*–TGF-β signaling pathway in regulation of the EMT and development of metastatic CRPC under ADT or AR-inhibitor conditions [118]. In that study, an extracellular matrix (ECM) component-secreted protein, TGF-β-induced (*TGFBI*), was shown to be associated with PCa EMT and activation in androgen-deprived PCa cells with a variety of metastatic properties. Gain of *TGFBI* function in vitro or in vivo revealed a significant correlation with EMT markers measured by p-Smad2 and vimentin. Knockdown of the AR in AR-positive cells enhanced *TGFBI* expression, and ectopic expression of the AR in AR-negative cells reduced *TGFBI* levels. Based on a gene set enrichment analysis (GSEA), Chen et al. identified the *SPDEF* gene as the upstream mediator between AR signaling and *TGFBI* expression. A clinical study using IHC reveled an inverse correlation between *SPDEF* and *TGFBI* in PCa before or after ADT. With loss of *SPDEF* function, TGF-β expression was retained, while ectopic expression of *SPDEF* decreased *TGFBI* in PCa cells. That study was the first to elucidate a role of the TF in the AR–TGF-β axis, which could provide another explanation for the common clinical problem of metastatic CRPC developing after a standard ADT regimen in PCa patients. Targeting the AR–*SPDEF*–*TGFBI* pathway could prevent further progression to metastatic CRPC (Figure 1).

## 7. E26 Transformation-Specific Variant 6 Gene (*ETV6*)

*ETV6*, also known as *TEL*, is a transcription repressor that belongs to the ETS TF gene family, located on chromosome 12p13 with eight exons and encoding a 452-amino acid protein. There are two domains present in *ETV6*: the ETS domain, which mediates DNA binding to the GGAA-rich region and is encoded by 6th and 7th exons, and the pointed (PNT) domain for protein–protein interactions, which is encoded by the 3rd and 4th exons [139,140]. *ETV6* translocation was reported in several types of cancers: in mesoblastic nephromas, congenital fibrosarcomas, human secretory breast carcinoma, and secretory carcinoma of the salivary gland [141,142,143,144,145]. *ETV6* plays a vital role in hematopoiesis and embryonic development.

In hematological malignancies, translocation at chromosome 12p13 is most commonly observed and involves *ETV6* and more than 25 partner genes, which have been molecularly characterized [146]. There are five potential mechanisms of *ETV6*-mediated leukemogenesis that have been identified: (1) constitutive activation of the kinase activity of the partner protein, (2) change in the original functions of a TF, (3) loss of function of a fusion gene, (4) activation of a proto-oncogene in the vicinity of a chromosomal translocation, and (5) a dominant negative effect of a fusion protein resulting from transcriptional repression mediated by wild-type *ETV6* [147]. The *ETV6*–*RUNX1* fusion protein is present in 25% of pediatric patients with B-cell precursor acute lymphoblastic leukemia [148], and this fusion was observed to be significant in modulating the proliferation and survival in vitro [149,150] and in vivo [151]. *LPXN* was reported to be a fusion partner gene at exon 2 with exon 6 in *ETV6* in relapsed acute myeloid leukemia (AML), and it played a crucial role in the enhanced proliferation response of the 32D myeloid cell line to granulocyte colony-stimulating factor (G-CSF) and C-X-C motif chemokine 12 (CXCL12) [152].

In PCa, a number of reports demonstrated that gene fusion between transmembrane protease serine 2 (*TMPRSS2*) and ETS occurred in more than 50% of patients [153,154]. Further reports revealed fusion of the *TMPRSS2* region (21q22.2) with ETS members, ERG at 21q22.2 and *ETV1* at 7q21.2 [155] or *ETV4* [156], indicating a mechanism for the overexpression of ETS in PCa. However, translocation of *ETV6* and *TMPRSS2* in PCa has not been reported. Frequent deletions and mutations of *ETV6* in advanced and metastatic PCa were observed [157,158].

In PCa, mutational analysis of *ETV6* in prostate carcinoma cell lines, xenografts, and metastatic foci revealed that an inactive protein might be produced that acts as a tumor-suppressor gene [159]. Tsai et al. showed that downregulation of *ETV6* through activation of an epidermal growth factor receptor (EGFR) signaling-mediated increase in *miR-96* expression contributed to PCa development based on in vitro and in vivo analyses [160]. A more recent study by Tsi et al., using a panel of mouse-derived prostate cancer cell lines and human prostate cancer, demonstrated that *ETV6* expression was negatively associated with *TWIST1*. Furthermore, the researchers identified one *ETV6* response element that was sequence-specific for the *TWIST1* promoter, suggesting the inhibition of EMT activation by repression of *TWIST1’s* transcriptional activity. In addition, disruption of *ETV6* promotes EGFR-tyrosine kinase inhibitor (TKI) resistance through perturbation of the *ETV6*–*TWIST1* axis by derepression of both *TWIST1* and EGFR–RAS signaling in an animal model [161].

**Figure 2:**
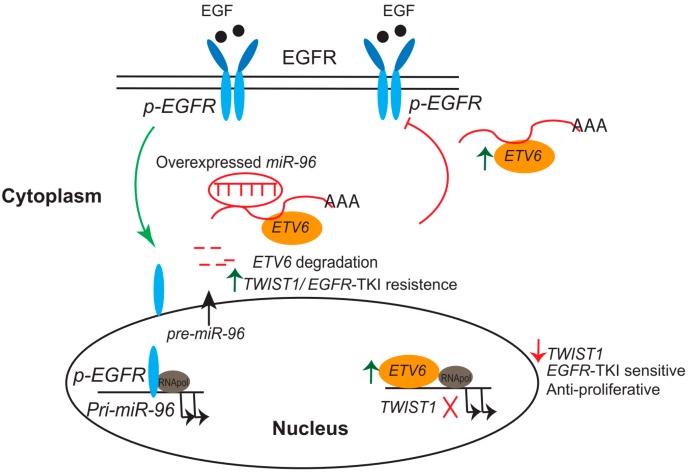
Proposed model for *TWIST1*/epidermal growth factor receptor tyrosine kinase inhibitor (EGFR-TKI) resistance and tumor progression. Degradation of *ETV6* through activation of EGFR signaling-mediated overexpression of *miR-96* contributes to tumor progression and activates drug resistance as a result of *TWIST1* expression (**Left**). Overexpression of *ETV6* inhibits EGFR activation and acts as a transcriptional repressor for *TWIST1* (**Right**).

These data support the hypothesis that prostatic metastasis involves EGFR signaling-mediated *miR-96* induction and *ETV6* inactivation, resulting in activation of *TWIST1*-stimulated metastatic phenotypes. Those studies proposed a novel function for *ETV6,* in which it acted as a central regulatory axis that connected EGFR signaling activation, and it may act as a potential marker for predicting single-agent therapy using EGFR-based anticancer therapy (Figure 2).

## 8. Future Directions

Although new biomarkers for PCa screening and diagnosis continue to be identified, current efforts should be focused on tracing the line between the early stage of primary PCa through metastatic CRPC, which occurs in 50% of PCa patients and remains incurable and lethal. Recently, several proposed mechanisms at the molecular level were revealed that suggested targeting both AR signaling by known ADT and suppressing downstream targets, which were shown to be responsible for activation of the EMT or metastatic CRPC after ADT. Previously, AR overexpression and AR mutations were identified as the main mechanisms for resistance to AR therapies. Most PCa cells express the wild-type AR gene, and AR mutations frequently occur in the late stage of PCa. One of the mechanisms for the failure of ADT is the presence of mutations in the AR that enhance AR’s activity in response to antiandrogen drugs. Also, these mutations can enhance AR’s transactivation and exert resistance to drugs such as the F876L point mutation to enzalutamide. Furthermore, an AR coactivator may activate the wild-type or mutant AR in response to adrenal androgen, which can progress to CRPC.

In hematological malignancies, molecular cytogenetics or DNA sequencing of blood or bone marrow samples are often used in the clinic to investigate *ETV6* mutations and chromosomal abnormalities [162,163]. However, in CRPC patients or before treating patients with TKIs, genetic mutations or expression levels of the *ETV6* gene at the mRNA or protein level are recommended using blood samples or by taking tissue biopsies for IHC staining. However, analyzing translocations of the *ETV6* gene in PCa by karyotyping has not been reported. In earlier stages of PCa, patients may still have a wild-type form of *ETV6* and drug sensitivity to TKIs. Therefore, earlier screening for *ETV6* is highly recommended.

Furthermore, measuring the mRNA levels of the *SPDEF* or *ZBTB46* genes in blood samples or their protein expressions using biopsies from PCa patients by IHC staining could help in tracking the progression of PCa from primary to CRPC or monitoring the efficiency of treatment with ADT.

Nowadays, several potential targets for patients with CRPC—like the AR-NTD inhibitor, EPI-002; the oral PARP inhibitor, olaparib; and the PI3K/mTOR inhibitor, BEZ235—have provided improvements in clinical outcomes. However, most patients still progress to an incurable stage. Understanding the AR oncogenic networks may provide a new strategy for diagnosis or therapeutic targets of primary and CRPC, which could improve PCa management and reduce high levels of morbidity and mortality. Inhibition of the AR by manipulating the ligand could provide additive therapeutic benefits. Modulation of intracellular pathways, such as kinases or nonkinases, with the ablation of androgen may improve clinical outcomes and delay PCa development.

While the mechanism of CRPC is still under investigation, one of the main drivers is the AR signaling pathway. There is a big challenge to identify suitable therapies for those patients with multiple aberrant AR signaling. In addition to patients with several point mutations or AR variants, coactivators that also play important roles in AR activity could be targeted. Accumulating evidence suggests that combinatorial therapies are recommended to overcome this disease.
