# Correction: Fararjeh, A-F S., et al. ZBTB46, SPDEF, ETV6 Novel Potential Biomarkers and Therapeutic Targets in Castration Resistance Prostate Cancer. *Int. J. Mol. Sci.* 2019, *20*, 2802

**DOI:** 10.3390/ijms21124243

**Published:** 2020-06-14

**Authors:** Abdul-Fattah Salah Fararjeh, Yen-Nien Liu

**Affiliations:** 1 Department of Medical Laboratory Sciences, Faculty of Science, Al-Balqa Applied University, Al-Salt 19117, Jordan; Fararjehas@bau.edu.jo; 2 Ph.D. Program for Cancer Molecular Biology and Drug Discovery, College of Medical Science and Technology, Taipei Medical University, Taipei 11031, Taiwan; 3 Graduate Institute of Cancer Biology and Drug Discovery, College of Medical Science and Technology, Taipei Medical University, Taipei 11031, Taiwan

The authors wish to make the following correction to this paper [1]: PSA, which was described as being initiated in “1979”, was actually initiated in “1970”.

The authors would like to apologize for any inconvenience caused to the readers by this change.
